# Effects of Grain Boundary Misorientation Angle on the Mechanical Behavior of Al Bicrystals

**DOI:** 10.3390/nano13233031

**Published:** 2023-11-27

**Authors:** Wilmer Velilla-Díaz, Habib R. Zambrano

**Affiliations:** 1 Instituto de Diseño y Métodos Industriales, Universidad Austral de Chile, Valdivia 5110566, Chile; 2 Departamento de Ingeniería Mecánica, Universidad del Norte, Barranquilla 081007, Colombia

**Keywords:** grain boundary effect, tilt GB, twist GB, fracture resistance, *CTOD*, *J*-integral, brittle–ductile behavior

## Abstract

This research article explores the effect of grain boundary (GB) misorientation on the mechanical behavior of aluminum (Al) bicrystals by means of molecular dynamics (MD) simulations. The effect of GB misorientation on the mechanical properties, fracture resistance, and crack propagation are evaluated under monotonic and cyclic load conditions. The *J*-integral and the crack tip opening displacement (CTOD) are assessed to establish the effect of the GB misorientation angle on the fracture resistance. The simulations reveal that the misorientation angle plays a significant role in the mechanical response of Al bicrystals. The results also evidence a gradual change in the mechanical behavior from brittle to ductile as the misorientation angle is increased.

## 1. Introduction

Tensile and fatigue resistance of metallic materials greatly depends on grain size and the effect of the misorientation angle on the propagation of tiny and sharp defects [1,2,3,4,5,6,7]. In order to improve mechanical and fatigue properties, the interaction between defects and GB has to be investigated. Previous experimental and computational investigations have established that the GB works as a barrier that blunts the tip and arrests the propagation of small cracks [3,6,7]. In nanocrystals of aluminum (Al), the volume fraction of GBs significantly affects the mechanical response of the material [8]. Single crystals normally show brittle behavior, whereas bicrystals tend to be ductile due to the presence of the GB [6,7]. On the other hand, considering that the relative grain misorientation angle plays an important role in the tensile and crack propagation resistances of crystalline materials [5,6,7,9,10,11,12,13,14], the effect of this angle is another factor that has to be deeply studied for a better understanding of some failure mechanics that occur in metallic materials, such as ductile, brittle and fatigue failure. The effect of the GB on the fatigue life has been investigated for polycrystals by means of nanomechanical experiments to improve the mechanical response under fatigue loads [15]. In order to simulate the interaction between an atomistic defect within a grain and a GB with a determined misorientation angle, some computational methods have successfully been utilized [16,17]. Molecular dynamics (MD) simulations are widely used to study the tensile response, deformation mechanisms and crack propagation of nanocrystalline materials [16,18,19,20,21,22]. Using MD, it has been found that the accumulation of dislocations at the crack tip reduces the crack propagation [19]. In nanocrystalline Cu, the effect of the thickness of amorphous films (AIFs) on crack propagation has also been analyzed by means of MD, finding that the films have a higher resistance to crack propagation when the AIF thickness increases [23]. MD has also brrn used to investigate the mechanisms of crack nucleation near to the crack tip in nanocrystalline materials, and the results show that crack nucleation occurs preferentially at triple junctions to release strain energy [24]. In crystalline materials, to characterize and quantify the crack propagation resistance, different fracture mechanics parameters are utilized, such as the *J*-integral (*J*), stress intensity factor (*K*) and crack tip opening displacement (CTOD) [25,26,27,28]. In the present research, MD simulations of nano-cracked Al bicrystals under monotonic and increasing cyclic load conditions are performed to study the effect of different grain misorientation angles on the deformation mechanisms, mechanical properties and fracture resistance. High and low, and tilt and twist GB misorientation angles are also considered to investigate the crack propagation phenomenon in Al bicrystals. The crack propagation resistance is assessed based on the elastoplastic fracture mechanic parameters *J* and CTOD, where CTOD is measured directly from MD simulations. The results show that by applying monotonic or increasing cyclic loading during the simulations, the mechanical behavior of the crystals is similar. However, using cyclic loading, the fracture process can be observed in more detail. In the case of Al bicrystals, different deformation mechanisms and fracture behavior are observed for the selected GB misorientations. Ductile and brittle behavior is observed in the simulations depending on the selected misorientation angle.

## 2. Materials and Methods

### 2.1. Molecular Dynamics Modeling

**Figure 1:**
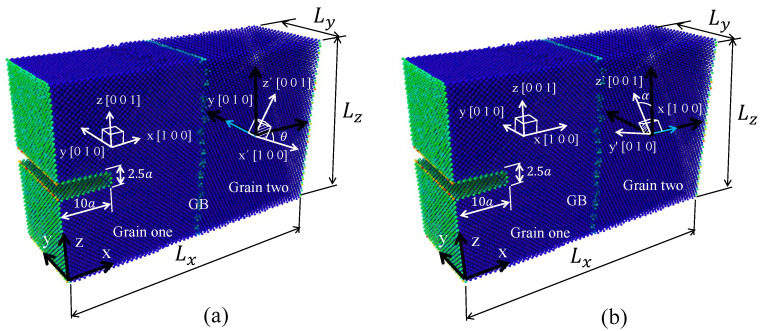
Atomistic system angle definition: (**a**) tilt GB (**b**) twist GB.

**Figure 2:**
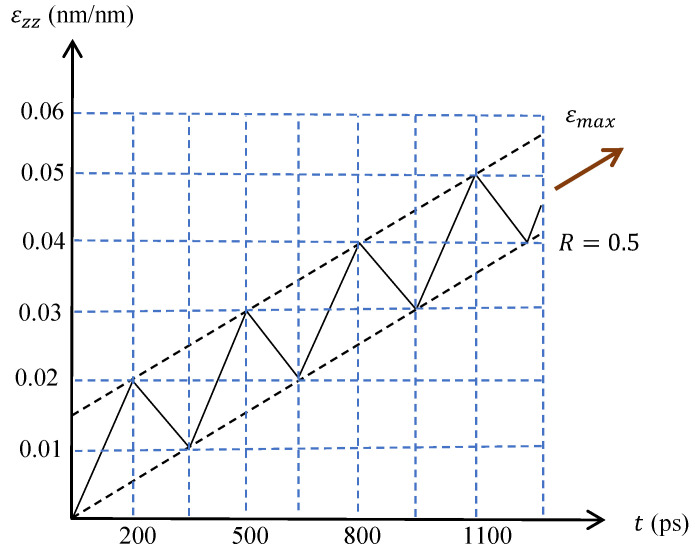
Cyclic loading applied during the simulations.

MD simulations were performed using the open-source code Large-scale Atomic Molecular Massively (LAMMPS) [29]. The embedded atom method (EAM) potential of Mendelev et al. [30] was selected for the MD simulations as it has in other research involving interaction defects with free surface and GBs [7,18,31,32]. To study the effect of the misorientation angle between two grains on the tensile and crack propagation resistances of Al crystals, 26 atomistic systems were simulated and the results were analyzed. The atomistic systems, grain misorientations and test conditions are specified in Table 1. θ is the angle that defines the tilt GB misorientation by rotating the second grain along the axis y=[010], as presented in Figure 1a. α defines the twist GB misorientation by rotating the second grain along the axis x=[100], as shown in Figure 1b. The width ($L_{x}$), thickness ($L_{y}$) and height ($L_{z}$) of the atomistic box are given by 60a×20a×40a, respectively, where *a* is the Al lattice, viz., a=0.405 nm. Each atomistic system contains 200,000 particles, and the initial edge crack geometry is presented in Figure 1a,b, which is located at 20a from the bottom of the system. For different atomistic arrangements, the conjugate gradient method was used to minimize the energy system, and periodic boundary conditions were considered in the directions [0 1 0] and [0 0 1]. In addition, an isobaric–isothermal ensemble was used to equilibrate the system at 300K and 1.01 bar for 20,000 timesteps of 0.001 ps using the Nose–Hoover barostat and thermostat according to [6]. The applied monotonic deformation was implemented based on [25] for the single crystal (SC) and bicrystal Al. For the cyclic loading simulations, the load was applied in deformation control according to Figure 2, using a strain rate of $1\times 10^{-4}$/ps.

### 2.2. Simulations and Specimens

**Figure 3:**
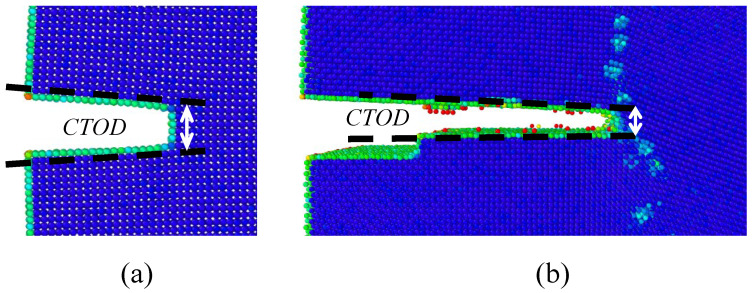
CTOD estimation for the (**a**) first grain and (**b**) second grain.

In order to study the mechanical properties and the crack propagation resistance of Al crystals with different grain misorientations, the atomistic systems which are summarized in Table 1 were modeled. Two systems without GBs (single crystals) and 24 systems with GBs (bicrystals) were used to study the effect of four different misorientation angles. Based on the simulations, the elastic module (*E*) and $S_{ut}$ were established using only smooth atomistic systems which were loaded monotonically (eight systems). Regarding the fracture resistance of cracked crystals, the $S_{ut}$ of cracked Al bicrystals was established for 16 cracked atomistic systems, and the results were compared with the SC. These 16 cracked atomistic systems with different misorientation GB were loaded as follows: eight of them using monotonic load, and increasing cyclic load on the other eight. It is worth pointing out that by implementing an increasing cyclic load in the simulations, the results allow us to observe the crack propagation in detail and the fracture mechanism. However, the effect of applying increasing cyclic load instead of monotonic load was also studied. On the other hand, CTOD and *J* were estimated to assess the crack propagation resistance. The maximum values of these two parameters ($CTOD_{U}$ and $J_{U}$) were obtained for different misorientation angles and compared. $J_{U}$ was obtained as defined in [33] as follows:

(1) $$J_{U}=\frac{\sigma \cdot CTOD_{U}\cdot \pi }{4}$$

where σ is the maximum global stress which is obtained in the simulation for both load conditions: monotonic and increasing cyclic load. The global stress was computed based on the Virial stress tensor [34] as follows:

(2) $$\sigma_{ij}=\frac{1}{V}\sum_{m=1}^{N}\left(\frac{1}{2}\sum_{m\neq n}^{N}F_{mn}^{i}r_{mn}^{j}-s_{m}v_{m}^{i}v_{m}^{j}\right)$$

where $\sigma_{ij}$ is the virial stress tensor, *V* is the atomistic system volume, $F_{mn}^{i}$ is the force vector between atom *m* and atom *n*, $r_{mn}^{j}$ is the distance vector between atom *m* and atom *n*, $s_{m}$ is the mass of the atom *m*, $v_{m}^{i}$ is the velocity vector of the atom *m* and *N* is the total number of atoms of the atomistic system. The deviation of the volume box due to the atoms in the free surfaces was corrected by using VORO++ [35]. The distance between atoms at the crack tip to estimate the CTOD was estimated using OVITO [36], as shown in Figure 3.

To analyze the effect of the GB misorientation on the crack propagation, the crack growth is monitored from the MD simulation by means of OVITO. To study crack propagation, we employ the centrosymmetry parameter (CSP) method, which quantifies the degree of local lattice symmetry and identifies defects around the crack tip [37]. Additionally, dislocation analysis (DXA) is utilized to track and analyze dislocation structures associated with crack propagation [38].

## 3. Results and Discussion

### 3.1. Deformation Mechanisms and Mechanical Properties

**Figure 4:**
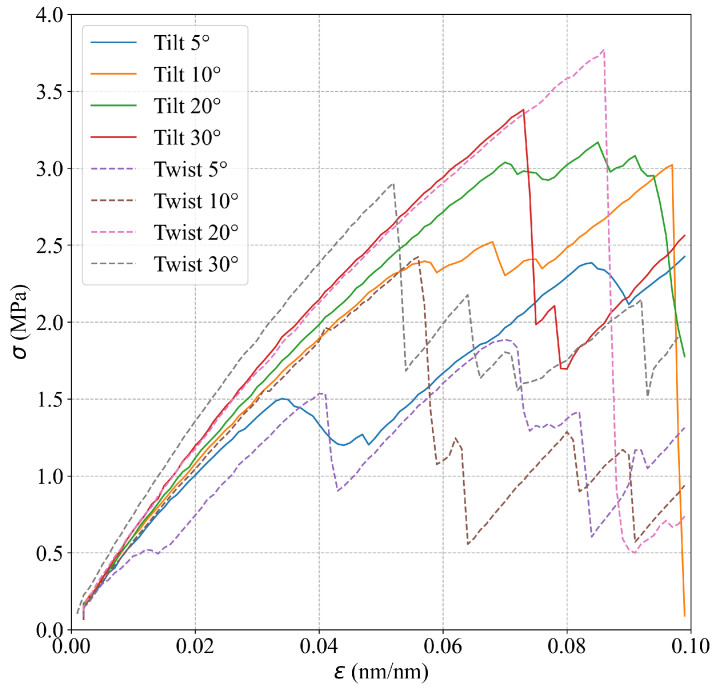
Effect of the grain misorientation on the stress–strain curves.

**Figure 5:**
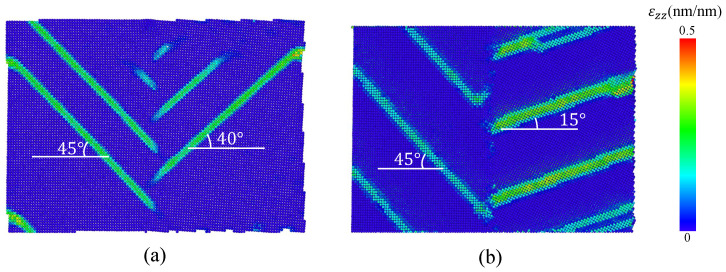
Strain in the *z*-direction for uncracked bicrystals with (**a**) θ=5° at ε=4.5% and (**b**) θ=30° at ε=8%.

The simulations show that the mechanical behavior of Al bicrystals within the elastic region is not affected significantly by the grain misorientation. Table 2 presents *E* values for different θ and α, which are estimated using the results from the smooth atomistic systems. In addition, Figure 4 shows the respective stress–strain curves for the different GB misorientations, where similar slopes for the linear elastic region for the different misorientations including tilt and twist angles are observed. Analyzing the strain in the *z*-direction, the slip planes that usually appear at 45° from the maximum principal stress are rotated in the simulations according to the corresponding misorientation angle. For example, for θ=5° and θ=30°, the slip planes in the second grain appear at 40° and 15°, respectively, as shown in Figure 5. For $S_{ut}$, the simulations show a marked effect of the misorientation angle on this property. Table 2 summarizes the $S_{ut}$ obtained for each misorientation angle. Figure 4 shows that $S_{ut}$ increases as the tilt angle grows, obtaining a maximum $S_{ut}$ at θ=30°. Regarding the simulations for twist angle misorientation, the results show that a final fracture is not evidenced in the simulations. However, the presence of many slip bands and a significant global stress drop (as seen in Figure 4) demonstrate that the material has failed. Figure 4 indicates that $S_{ut}$ reaches a maximum value for the twist angle at α=20°.

### 3.2. Fracture Resistance

**Figure 6:**
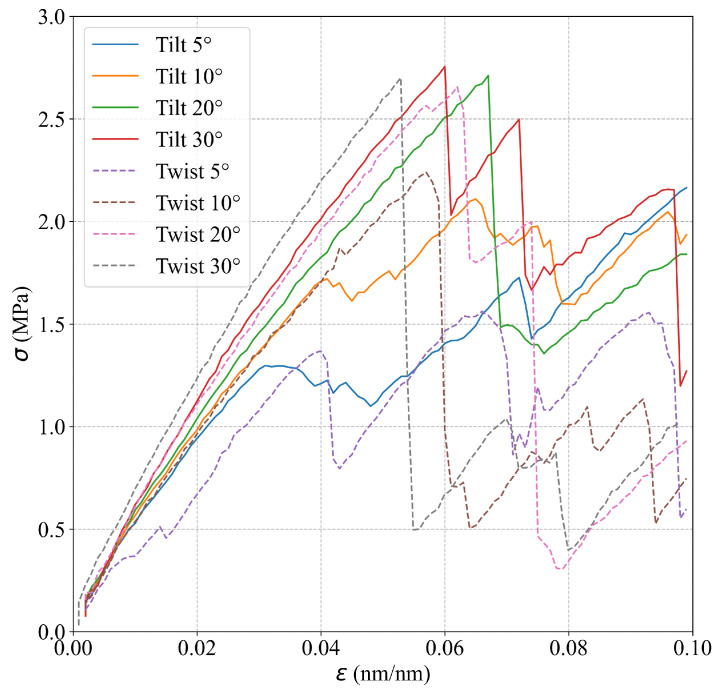
Stress–strain curves for cracked atomistic systems with different GB misorientations.

**Figure 7:**
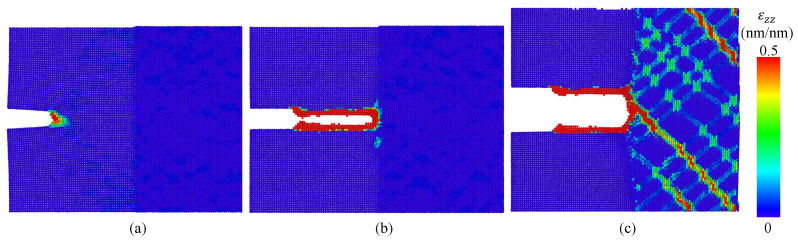
Strain in the *z*-direction for α=20° at (**a**) $\varepsilon_{zz}=6\%$, (**b**) $\varepsilon_{zz}=6.5\%$ and (**c**) $\varepsilon_{zz}=20\%$.

**Figure 8:**
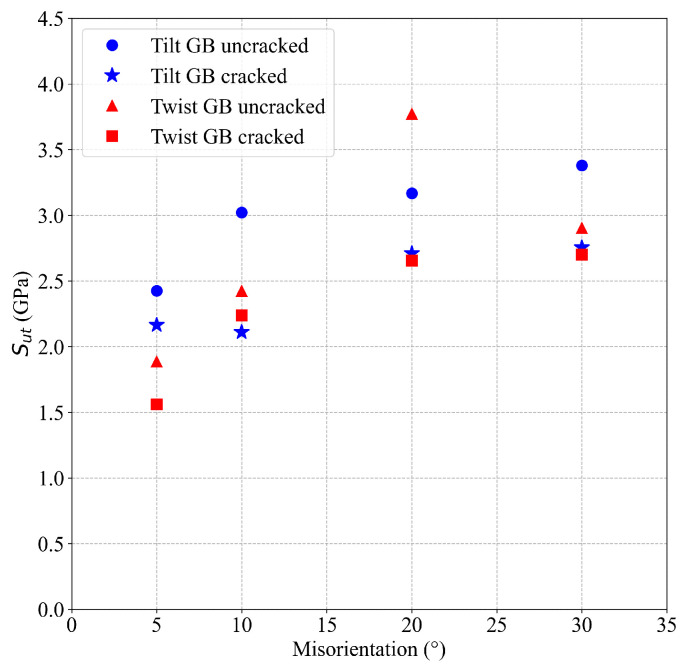
Effect of grain misorientation on the $S_{ut}$ of Al bicrystals.

**Figure 9:**
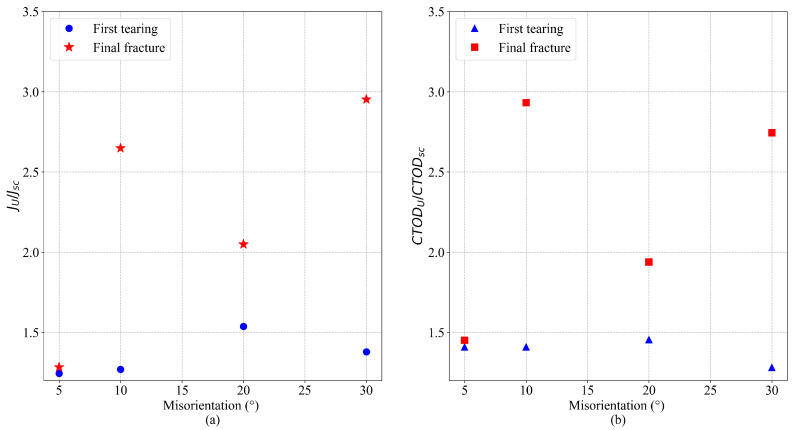
Effect of tilt GB misorientation on (**a**) $J_{U}$ and (**b**) $CTOD_{U}$ for Al bicrystals.

A significant effect of the GB misorientation on the stress–strain curves of cracked Al bicrystals is observed in the simulations. Figure 6 shows the stress–strain curves for cracked Al bicrystals for different GB misorientation angles. Due to the interaction between the GB and the crack tip, small crack increments before the final fracture of cracked atomistic systems (tearing) are observed during the simulations of Al bicrystals with tilt misorientation. For twist misorientation, no crack propagation at all is evidenced for α=5° and α= 10°; instead, a quasi-ductile failure takes place. For α=20° and α= 30°, a first tearing is observed, which is arrested by the GB, and it is followed by a quasi-ductile failure (see Figure 7). A comparison of the effect of the misorientation angle (θ and α) on the $S_{ut}$ of uncracked and cracked crystals (obtained from Figure 4 and Figure 6, respectively) is shown in Figure 8. This figure evidences that $S_{ut}$ for cracked Al bicrystals increases with θ, and the maximum value is obtained at θ= 30°. For twist misorientation, the maximum value is reached at α=20°. Analysing the $S_{ut}$ for uncracked and cracked bicrystals, Figure 8 reveals that the misorientation angles that yield the largest drop on the $S_{ut}$ due to the presence of an initial crack are θ=10° and α=20°, respectively. $S_{ut}$ decreases by 23.5% for θ=10°, and 30% for α=20°. Regarding the fracture mechanics analysis, the effects of the misorientation angle on $CTOD_{U}$ and $J_{U}$ are presented in Figure 9, where the results are normalized by $CTOD_{sc}$ and $J_{sc}$ which correspond to the fracture toughness of the SC obtained from [25]. $CTOD_{U}$, $J_{U}$ and the variables used to compute $J_{U}$ are summarized in Table 3. It is worth pointing out that ductile behavior is observed in all of the cracked atomistic systems with twist misorientation angles. Therefore, the results were not analyzed by means of fracture mechanics for these atomistic systems. The values of CTOD and *J* are estimated just at the beginning of the first tearing, viz., $CTOD_{ft}$ and $J_{ft}$, respectively, and they also are reported in Table 3.

### 3.3. Crack Propagation under Cyclic Loading

**Figure 10:**
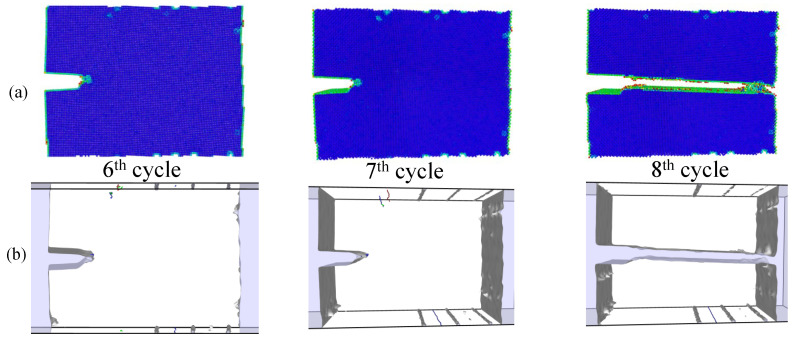
Local defect analysis for θ= 5° (**a**) by CPS and (**b**) by DXA.

**Figure 11:**
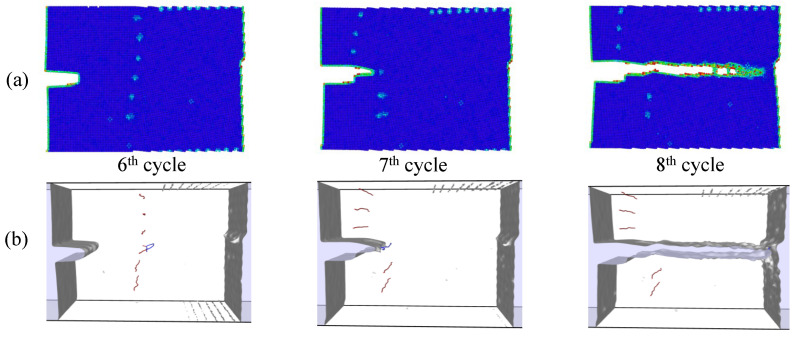
Local defect analysis for θ= 10° (**a**) by CPS and (**b**) by DXA.

**Figure 12:**
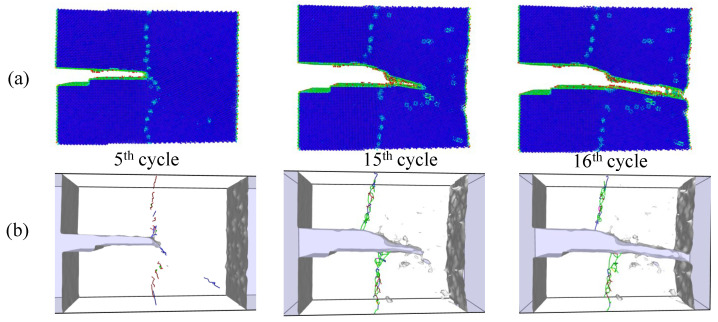
Local defect analysis for θ= 20° (**a**) by CPS and (**b**) by DXA.

**Figure 13:**
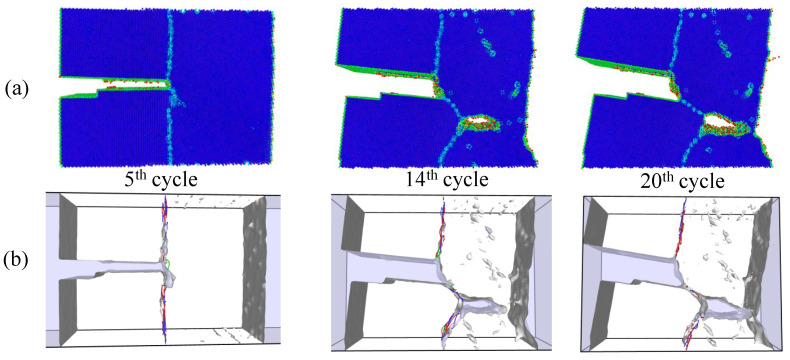
Local defect analysis for θ= 30° (**a**) by CPS and (**b**) by DXA.

**Figure 14:**
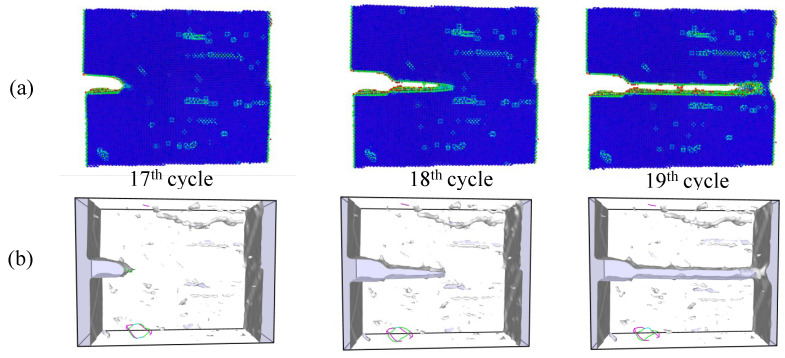
Local defect analysis for α= 5° (**a**) by CPS and (**b**) by DXA.

**Figure 15:**
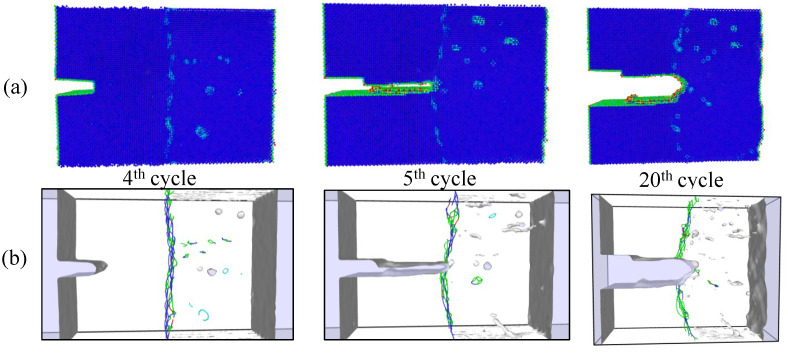
Local defect analysis for α= 10° (**a**) by CPS and (**b**) by DXA.

**Figure 16:**
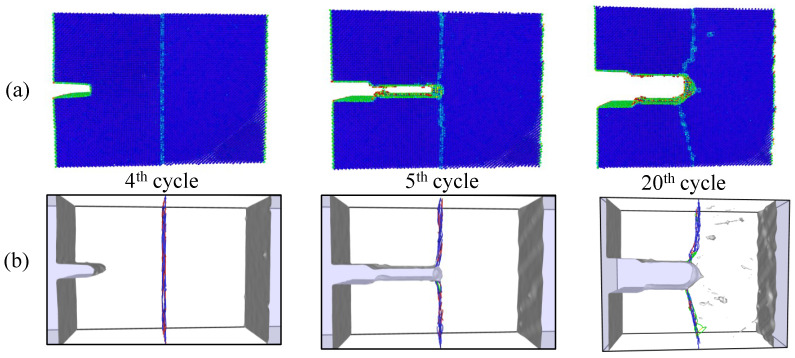
Local defect analysis for α= 20° (**a**) by CPS and (**b**) by DXA.

**Figure 17:**
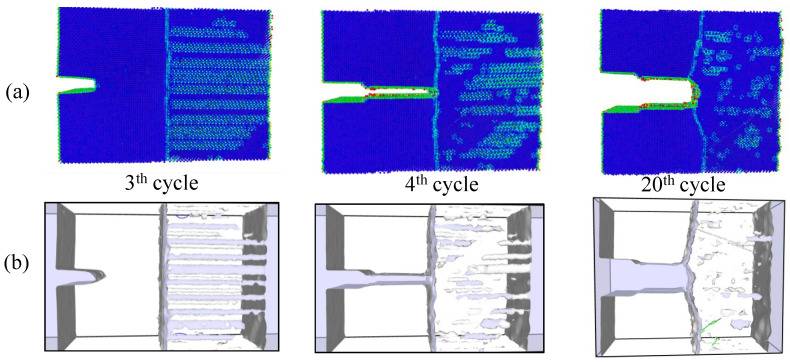
Local defect analysis for α= 30° (**a**) by CPS and (**b**) by DXA.

**Figure 18:**
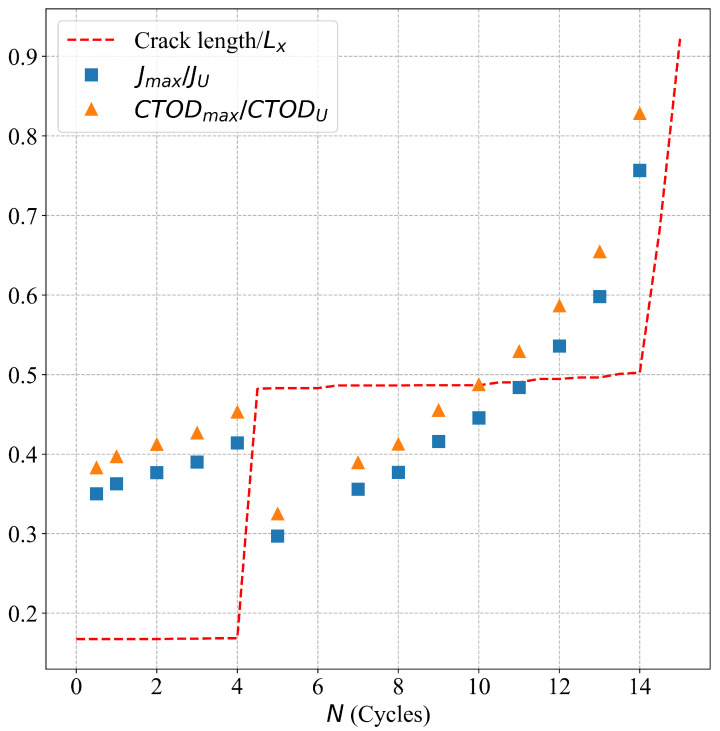
Fracture process for θ= 20° under cyclic loads.

Simulations under cyclic loading conditions allow us to observe the fracture process in detail and to analyze the mechanical behavior of bicrystals with different grain misorientations. For tilt GBs, the results show brittle crack propagation for θ=5° and θ=10°, a mix mode (fracture between ductile and brittle) for θ=20° and ductile behavior for θ=30°, as seen in Figure 10, Figure 11, Figure 12 and Figure 13, respectively. Tearing is evidenced in Figure 11, Figure 12 and Figure 13 for θ=10°, θ=20° and θ=30°. Regarding bicrystals with twist misorientation, several vacancies and dislocations are observed in Figure 14, Figure 15, Figure 16 and Figure 17, which indicate a ductile behavior during failure. In addition, tearing also appears in the simulations for twist angles, as shown in Figure 14, Figure 15, Figure 16 and Figure 17. The crack growth cycle by cycle is analyzed in Figure 18 for θ= 20°. This analysis for (θ= 20°) shows how the maximum CTOD$\left(CTOD_{max}\right)$ and $J(J_{max})$ increase each cycle until the atomistic system is broken and the crack length grows every cycle. Figure 18 evidences that the $CTOD_{max}$ and $J_{max}$ grow steadily cycle by cycle until the fourth cycle, where tearing begins in the first grain, while $CTOD_{max}$ and $J_{max}$ drop with the crack growth, then the crack is arrested by the GB. After the crack arresting, $CTOD_{max}$ and $J_{max}$ increase again steadily until the final fracture occurs at the 16-th cycle.

## 4. Conclusions

The effect of the GB misorientation angle on the mechanical behavior of Al bicrystals is evidenced throughout the present article by means of molecular dynamic simulations. The following main conclusions are derived from this research work:

- GB misorientation has a beneficial effect on the mechanical properties of Al bicrystals, increasing the $S_{ut}$ with increasing misorientation angles.
- Regarding fracture resistance, the GB misorientation improves $CTOD_{U}$ and $J_{U}$ with increasing misorientation angles.
- A gradual change from brittle (for 0° ≤θ<20°) to ductile (for θ > 20°) behavior is observed in Al bicrystals for tilt GB misorientations.
- For twist GB misorientations, abundant voids and dislocations are formed in the bicrystals, suggesting a ductile behavior.
- GB misorientation works as a barrier that arrests crack growth, as observed in the simulations of cracked bicrystals.

## Figures and Tables

**Table 1:** Atomistic system configurations.

Atomistic System	Loading	Initial Defect	GB	Angle (°)
Single crystal	Monotonic	Edge crack	-	-
	Cyclic	Edge crack	-	-
Bicrystal	Monotonic	Edge crack	Tilt (θ)	5, 10, 20, 30
	Monotonic	Edge crack	Twist (α)	5, 10, 20, 30
	Monotonic	-	Tilt (θ)	5, 10, 20, 30
	Monotonic	-	Twist (α)	5, 10, 20, 30
	Cyclic	Edge crack	Tilt (θ)	5, 10, 20, 30
	Cyclic	Edge crack	Twist (α)	5, 10, 20, 30

**Table 2:** Mechanical properties of the aluminum bicrystal with different misorientation angles.

GB	Angle (°)	*E* (GPa)	$S_{\mathrm{ut}}$ (GPa)
Tilt	5	59.72	2.4200
	10	61.20	3.0211
	20	62.06	3.1683
	30	61.30	3.3804
Twist	5	64.33	1.8857
	10	65.25	2.4225
	20	69.87	3.7715
	30	67.50	2.9035

**Table 3:** Fracture resistance for different misorientation angles.

	Angle (°)	${\mathrm{CTOD}}_{\mathrm{ft}}$ (nm)	${\mathrm{CTOD}}_{U}$ (nm)	σ (GPa)	$J_{\mathrm{ft}}$ (J/m^2^)	$J_{U}$ (J/m^2^)
Tilt GB	5	1.987	2.045	2.267	3.53	3.64
	10	1.987	4.133	2.313	3.61	7.51
	20	2.052	2.733	2.711	4.36	5.81
	30	1.807	3.870	2.755	3.91	8.37
Twist GB	5	-	-	1.561	-	-
	10	-	-	2.239	-	-
	20	1.406	-	2.655	2.93	-
	30	1.533	-	2.700	3.25	-

## Data Availability

Data are contained within the present article.

## References

[1] Berman D., Krim J. (2013). Surface science, MEMS and NEMS: Progress and opportunities for surface science research performed on, or by, microdevices. Prog. Surf. Sci..

[2] Li X., Bhushan B. (2003). Fatigue studies of nanoscale structures for MEMS/NEMS applications using nanoindentation technique. Surf. Coatings Technol..

[3] Wang B., An X., Xue P., Liu F., Ni D., Xiao B., Liu Y., Ma Z. (2023). Grain size effects on high cycle fatigue behaviors of pure aluminum. Int. J. Fatigue.

[4] Nellessen J., Sandlöbes S., Raabe D. (2016). Low cycle fatigue in aluminum single and bi-crystals: On the influence of crystal orientation. Mater. Sci. Eng. A.

[5] Xie G., Wang F., Lai X., Xu Z., Zeng X. (2023). Atomistic study on crystal orientation-dependent crack propagation and resultant microstructure anisotropy in NiTi alloys. Int. J. Mech. Sci..

[6] Velilla-Díaz W., Pacheco-Sanjuan A., Zambrano H.R. (2019). The role of the grain boundary in the fracture toughness of aluminum bicrystal. Comput. Mater. Sci..

[7] Velilla-Díaz W., Zambrano H.R. (2021). Crack length effect on the fracture behavior of single-crystals and bi-crystals of aluminum. Nanomaterials.

[8] Meyers M.A., Mishra A., Benson D.J. (2006). Mechanical properties of nanocrystalline materials. Prog. Mater. Sci..

[9] Chandra S., Kumar N.N., Samal M.K., Chavan V.M., Raghunathan S. (2017). An atomistic insight into the fracture behavior of bicrystal aluminum containing twist grain boundaries. Comput. Mater. Sci..

[10] Zhang Y., Jiang S., Zhu X., Zhao Y. (2016). A molecular dynamics study of intercrystalline crack propagation in nano-nickel bicrystal films with (0 1 0) twist boundary. Eng. Fract. Mech..

[11] Shu X.T., Xiao S., Ma L., Deng H. (2017). Atomistic simulation of crack propagation in single crystal tungsten under cyclic loading. J. Mater. Res..

[12] Ovid I.A. (2007). Review on the fracture processes in nanocrystalline materials. J. Mater. Sci..

[13] Wang Y., Fu R., Zhou X., Thompson G.B., Yu Z., Li Y. (2016). Enhanced mechanical properties of pure copper with a mixture microestructure of nanocrystalline and ultrafine grains. Mater. Lett..

[14] Chatterjee A., Sharma G., Varshney J., Neogy S., Singh R.N. (2017). Comparative study of mechanical properties of pure nanocrystalline Ni and Ni-Tf nanocomposite. Mater. Sci. Eng. A.

[15] Karami M., Zhu Z., Zeng Z., Tamura N., Yang Y., Chen X. (2020). Two-tier compatibility of superelastic bicrystal micropillar at grain boundary. Nano Lett..

[16] Hahn E.N., Meyers M.A. (2015). Grain-size dependent mechanical behavior of nanocrystalline metals. Mater. Sci. Eng. A.

[17] Haouala S., Segurado J., LLorca J. (2018). An analysis of the influence of grain size on the strength of FCC polycrystals by means of computational homogenization. Acta Mater..

[18] Horstemeyer M.F., Farkas D., Kim S., Tang T., Potirniche G. (2010). Nanostructurally small cracks (NSC): A review on atomistic modeling of fatigue. Int. J. Fatigue.

[19] White P. (2012). Molecular dynamic modelling of fatigue crack growth in aluminium using LEFM boundary conditions. Int. J. Fatigue.

[20] Brandenburg J.E., Barrales-Mora L.A., Tsurekawa S., Molodov D.A. (2023). Dynamic behavior of grain boundaries with misorientations in the vicinity of ∑ 3 coherent and incoherent twin boundaries in Al bicrystals. Acta Mater..

[21] Zhou S., Chen P., Zha M., Zhu Y., Li B., Wang H.Y. (2023). Sequential transmutation of prismatic dislocations during {11$\bar{2}$2} twin-slip interaction in titanium. Scr. Mater..

[22] Xue D., Wei W., Shi W., Zhou X.R., Wen S.P., Wu X.L., Gao K.Y., Rong L., Qi P., Huang H. (2023). Dislocation evolution and induced precipitation on corrosion resistance of a novel Al-Mg-Zn-Er-Zr alloy during hot compression. Rare Met..

[23] Pal S., Reddy K.V., Deng C. (2019). On the role of Cu-Zr amorphous intergranular films on crack growth retardation in nanocrystalline Cu during monotonic and cyclic loading conditions. Comput. Mater. Sci..

[24] Li X., Jiang X. (2019). Theoretical analyses of nanocrack nucleation near the main crack tip in nano and micro crystalline materials. Eng. Fract. Mech..

[25] Velilla-Díaz W., Ricardo L., Palencia A., Zambrano H.R. (2021). Fracture toughness estimation of single-crystal aluminum at nanoscale. Nanomaterials.

[26] Ding J., Zheng H.-R., Tian Y., Huang X., Song K., Lu S.-Q., Zeng X.-G., Ma W.-S. (2020). Multi-scale numerical simulation of fracture behavior of nickel-aluminum alloy by coupled molecular dynamics and cohesive finite element method (CFEM). Theor. Appl. Fract. Mech..

[27] Liu Q.Y., Zhou J., Bao J.D., Zhao Y.W., Xiong L.C., Shi T.L., Long Y.H. (2019). A semi-empirical fracture model for silicon cleavage fracture and its molecular dynamics study. Theor. Appl. Fract. Mech..

[28] Skogsrud J., Thaulow C. (2015). Application of CTOD in atomistic modeling of fracture. Eng. Fract. Mech..

[29] Plimpton S. (1995). Fast Parallel Algorithms for Short-Range Molecular Dynamics. J. Comput. Phys..

[30] Mendelev M.I., Kramer M.J., Becker C.A., Asta M. (2008). Analysis of semi-empirical interatomic potentials appropriate for simulation of crystalline and liquid Al and Cu. Philos. Mag..

[31] Chandra S., Samal M.K., Chavan V.M. (2019). Dislocation nucleation from damaged grain boundaries in face centered cubic metals—An atomistic study. Materialia.

[32] Ji H., Ren K., Ding L., Wang T., Li J.M., Yang J. (2022). Molecular dynamics simulation of the interaction between cracks in single-crystal aluminum. Mater. Today Commun..

[33] Anderson T.L. (2005). Fracture Mechanics.

[34] Thompson A.P., Plimpton S.J., Mattson W. (2009). General formulation of pressure and stress tensor for arbitrary many-body interaction potentials under periodic boundary conditions. J. Chem. Phys..

[35] Rycroft C.H. (2009). VORO ++: A three-dimensional Voronoi cell library in C++. Chaos.

[36] Stukowski A. (2010). Visualization and analysis of atomistic simulation data with OVITO—The Open Visualization Tool. Model. Simul. Mater. Sci. Eng..

[37] Kelchner C.L., Plimpton S.J., Hamilton J.C. (1998). Dislocation nucleation and defect structure during surface indentation. Phys. Rev. B.

[38] Stukowski A., Bulatov V.V., Arsenlis A. (2012). Automated identification and indexing of dislocations in crystal interfaces. Model. Simul. Mater. Sci. Eng..

